# Physiological characterization of formyl peptide receptor expressing cells in the mouse vomeronasal organ

**DOI:** 10.3389/fnana.2014.00134

**Published:** 2014-11-21

**Authors:** Tobias Ackels, Benoît von der Weid, Ivan Rodriguez, Marc Spehr

**Affiliations:** ^1^Department of Chemosensation, RWTH Aachen UniversityAachen, Germany; ^2^Department of Genetics and Evolution, University of GenevaGeneva, Switzerland

**Keywords:** vomeronasal receptor, formyl peptide receptor, vomeronasal organ, sensory neurons, VNO, olfaction

## Abstract

The mouse vomeronasal organ (VNO) is a chemosensory structure that detects both hetero- and conspecific social cues. Based on largely monogenic expression of either type 1 or 2 vomeronasal receptors (V1Rs/V2Rs) or members of the formyl peptide receptor (FPR) family, the vomeronasal sensory epithelium harbors at least three neuronal subpopulations. While various neurophysiological properties of both V1R- and V2R-expressing neurons have been described using genetically engineered mouse models, the basic biophysical characteristics of the more recently identified FPR-expressing vomeronasal neurons have not been studied. Here, we employ a transgenic mouse strain that coexpresses an enhanced variant of yellow fluorescent protein together with FPR-rs3 allowing to identify and analyze FPR-rs3-expressing neurons in acute VNO tissue slices. Single neuron electrophysiological recordings allow comparative characterization of the biophysical properties inherent to a prototypical member of the FPR-expressing subpopulation of VNO neurons. In this study, we provide an in-depth analysis of both passive and active membrane properties, including detailed characterization of several types of voltage-activated conductances and action potential discharge patterns, in fluorescently labeled vs. unmarked vomeronasal neurons. Our results reveal striking similarities in the basic (electro) physiological architecture of both transgene-expressing and non-expressing neurons, confirming the suitability of this genetically engineered mouse model for future studies addressing more specialized issues in vomeronasal FPR neurobiology.

## Introduction

For mammals, the sense of smell is crucial to interact adequately with their environment. Fundamental information about hetero- and conspecifics, such as identity, social or reproductive state, is gathered by the olfactory system. In most mammals, this system consists of up to four anatomically and functionally distinct subsystems: the main olfactory system (Firestein, 2001; Mombaerts, 2004), the Grueneberg ganglion (Fuss et al., 2005; Koos and Fraser, 2005; Roppolo et al., 2006; Brechbühl et al., 2008; Schmid et al., 2010), the septal organ of Masera (Adams, 1992; Ma et al., 2003) and the vomeronasal organ (VNO). The VNO is a bilateral tubular structure located at the base of the nasal septum. Vomeronasal sensory neurons (VSNs) are highly sensitive chemoreceptors thought to primarily detect semiochemicals and other social cues (Leinders-Zufall et al., 2000; Dulac and Torello, 2003; Spehr et al., 2006; Ferrero et al., 2013). VSNs project single unbranched axons to the accessory olfactory bulb (AOB). To date, members of at least four chemoreceptor gene families are expressed in VSNs: the *V1r* (Dulac and Axel, 1995) and *V2r* (Herrada and Dulac, 1997; Matsunami and Buck, 1997; Ryba and Tirindelli, 1997) families, with more than 100 functional members each, a few odorant receptors (Lévai et al., 2006), and the recently discovered formyl peptide receptor (FPR)-related sequence (*Fpr-rs*) family of putative chemoreceptor genes. The *Fpr-rs* family comprises 7 members, 5 of which (*Fpr-rs1*, *rs3, rs4, rs6* and* rs7*) are predominantly or exclusively expressed in subsets of VSNs (Liberles et al., 2009; Rivière et al., 2009; Chamero et al., 2012). As key mediators of leukocyte chemotaxis, FPR1 and FPR-rs2 receptor proteins are expressed in immune cells such as granulocytes and monocytes (Rivière et al., 2009; He et al., 2013) where they serve crucial functions in host defense against pathogens by detecting microbe- and/or host-derived inflammation-associated metabolites (Migeotte et al., 2006; Le et al., 2007; Soehnlein and Lindbom, 2010). However, neither FPR1 nor FPR-rs2 was found transcribed in mouse VSNs (Liberles et al., 2009; Rivière et al., 2009).

Vomeronasal sensory neurons expressing members of the V1R family of G protein-coupled receptors are located in the more apical part of the vomeronasal sensory epithelium. These neurons co-express the G-protein α-subunit Gα_i2_ and project to the anterior part of the AOB (Belluscio et al., 1999; Rodriguez et al., 1999). Functionally, V1R neurons respond to sulfated steroids and to a variety of other secreted ethologically relevant small semiochemicals (Leinders-Zufall et al., 2000; Boschat et al., 2002; Novotny, 2003; Nodari et al., 2008; Isogai et al., 2011). By contrast, V2R expression is restricted to VSNs in the more basal Gα_o_-positive layer (Martini et al., 2001; Matsuoka et al., 2001; Dulac and Torello, 2003). V2R neurons predominantly detect peptides/small proteins (Leinders-Zufall et al., 2004; Chamero et al., 2007; Kimoto et al., 2007; Ferrero et al., 2013; Kaur et al., 2014) and project to the posterior region of the AOB. For FPR-rs3 expressing neurons, we recently described axonal projections to the rostral AOB (Dietschi et al., 2013), the target region of V1R neurons.

The single *Fpr-rs* gene cluster is adjacent to a stretch of more than 30 *V1/2r* genes. However, neither *V1rs*, nor *V2rs* share significant sequence homology with vomeronasal *Fpr-rs* genes. Liberles and coworkers suggested that vomeronasal *Fpr*s evolved from recent gene duplications and positive selection in the rodent lineage (Liberles et al., 2009). Together with recent functional data obtained from recombinant FPR expression (Bufe et al., 2012), these considerations argue for a neofunctionalization of vomeronasal *Fpr-rs* genes. Their predicted seven-transmembrane topology, their selective, punctate and monogenic vomeronasal expression pattern, and their localization in microvillous dendritic VSN endings (Liberles et al., 2009; Rivière et al., 2009), however, strongly suggest a functional role of FPR-rs in vomeronasal chemosignaling. Interestingly, while *Fpr-rs1* is coexpressed with G_αo_ in basal sensory neurons, the remaining vomeronasal *Fpr-rs* genes all coexpress G_αi2_ in the apical layer of the VNO neuroepithelium (Liberles et al., 2009; Munger, 2009; Rivière et al., 2009). Vomeronasal sensory neurons are activated *in situ* by formylated peptides and various other antimicrobial/inflammatory modulators (Rivière et al., 2009; Chamero et al., 2011) and heterologously expressed FPR-rs proteins retain agonist spectra that share some similarities to immune system FPRs (Rivière et al., 2009). However, the exact biological role of vomeronasal FPRs remains to be determined.

To address the neurobiological function of vomeronasal FPRs experimentally, a detailed physiological characterization of *Fpr-rs* neurons in their native environment is mandatory. Genetically modified animals in which the receptor identity of a given chemosensory neuron is marked by coexpression of a fluorescent reporter have proven particularly fruitful in the analysis of olfactory signaling (Boschat et al., 2002; Bozza et al., 2002; Grosmaitre et al., 2006, 2009; Oka et al., 2006; Ukhanov et al., 2007; Leinders-Zufall et al., 2009; Pacifico et al., 2012). Here, we describe a transgenic mouse strain that expresses FPR-rs3 together with a fluorescent marker (Fpr-rs3-i-Venus). This mouse model allows optical identification and subsequent physiological analysis of FPR-rs3-expressing neurons in acute VNO tissue slices. Using single neuron patch-clamp recordings, we thus provide an in-depth electrophysiological characterization of the basic biophysical properties inherent to a prototypical member of the FPR-expressing subpopulation of VNO neurons. Our analysis spans several types of voltage-activated conductances as well as action potential discharge parameters in both fluorescently labeled and control VSNs. Our data reveal a number of physiological similarities between FPR-rs3-expressing and non-expressing neurons. Together, these results confirm the suitability of Fpr-rs3-i-Venus mice for future studies of vomeronasal FPR neurobiology and, in addition, these findings indicate that the FPR expression does not confer a distinct biophysical phenotype to the subpopulation of FPR-positive VSNs.

## Materials and methods

### Animals

All animal procedures were in compliance with local and European Union legislation on the protection of animals used for experimental purposes (Directive 86/609/EEC) and with recommendations put forward by the Federation of European Laboratory Animal Science Associations (FELASA). Both C57BL/6 mice (Charles River Laboratories, Sulzfeld, Germany) and Fpr-rs3-i-Venus mice were housed in groups of both sexes at room temperature on a 12 h light/dark cycle with food and water available *ad libitum*. Experiments used young adults of either sex. We did not observe obvious gender-dependent differences.

### Transgenic mice

The transgene (Fpr-rs3-i-Venus) contains the FPR-rs3 coding sequence followed by an internal ribosome entry site (IRES), and the coding sequence for tau-Venus, a fusion between the microtubule-associated protein tau and Venus yellow fluorescent protein (Nagai et al., 2002). These coding sequences are under the control of the H element followed by the MOR28 promoter (Serizawa et al., 2006; modified by and generously provided by P. Feinstein). The Fpr-rs3-i-Venus transgene was isolated on gel after BssHII digestion and purified using the QIAquick^®^ Gel extraction kit (QIAGEN, Hilden, Germany). The transgene was injected into the pronuclei of fertilized C57BL6/DBA2 mouse oocytes following standard procedures. Four founders carrying the transgene were obtained. One of these founder animals expressed the transgene in VSNs and was, thus, used to start the colony. Backcrossed to C57BL/6J, mice were kept hemizygous. Wild type and transgenic mice had no obvious differences in size, weight, fertility, life expectancy or food consumption.

### Chemicals and solutions

The following solutions were used: (**S**_1_) 4-(2-Hydroxy-ethyl)piperazine-1-ethanesulfonic acid (HEPES) buffered extracellular solution containing (in mM) 145 NaCl, 5 KCl, 1 CaCl_2_, 1 MgCl_2_, 10 HEPES; pH = 7.3 (adjusted with NaOH); osmolarity = 300 mOsm (adjusted with glucose). (**S**_2_) Oxygenated (95% O_2_, 5% CO_2_) extracellular solution containing (in mM) 125 NaCl, 25 NaHCO_3_, 5 KCl, 1 CaCl_2_, 1 MgSO_4_, 5 BES; pH = 7.3; osmolarity = 300 mOsm. (**S**_3_) solution containing (in mM) 144 NaCl, 5 KCl, 1 TEACl, 1 CaCl_2_, 1 MgCl_2_, 10 HEPES, pH 7.3; osmolarity = 300 mOsm. (**S**_4_) solution containing (in mM) 134 NaCl, 5 KCl, 1 TEACl, 1 CaCl_2_, 1 MgCl_2_, 10 HEPES, 10 4-AP, pH 7.3; osmolarity = 300 mOsm. (**S**_5_) solution containing (in mM) 110 NaCl, 5 KCl, 25 TEACl, 1 CaCl_2_, 1 MgCl_2_, 10 HEPES, 10 4-AP, pH 7.3; osmolarity = 300 mOsm. (**S**_6_) solution containing (in mM) 115 NaCl, 25 TEACl, 1, 1 MgCl_2_, 10 HEPES, 10 4-AP, pH 7.3; osmolarity = 300 mOsm. (**S**_7_) solution containing (in mM) 105 NaCl, 25 TEACl, 5 mM BaCl_2_, 1 MgCl_2_, 10 HEPES, 10 4-AP, pH 7.3; osmolarity = 300 mOsm. (**S**_8_) Pipette solution containing (in mM) 143 KCl, 2 KOH, 1 EGTA, 0.3 CaCl_2_ (free Ca^2+^ = 110 nM), 10 HEPES, 2 MgATP, 1 NaGTP; pH = 7.1 (adjusted with KOH); osmolarity = 290 mOsm. (**S**_9_) Pipette solution containing (in mM) 133 CsCl, 10 NaCl, 2 CsOH, 1 EGTA, 0.3 CaCl_2_ (free Ca^2+^ = 110 nM), 10 HEPES, 1 MgATP, 1 NaGTP; pH = 7.1 (adjusted with CsOH); osmolarity = 290 mOsm.

Free Ca^2+^ and Mg^2+^ concentrations were calculated using WEBMAXC STANDARD^1^. If not stated otherwise, chemicals were purchased from Sigma (Schnelldorf, Germany). ω-agatoxin IVa and ω-conotoxin GVIA were purchased from Biotrend (Zurich, Switzerland). Stimuli and pharmacological agents were applied from air pressure-driven reservoirs via an 8-in-1 multi-barrel “perfusion pencil” (Science Products, Hofheim, Germany; Veitinger et al., 2011).

### Cryosections

For preparation of cryosections, the VNO was fixed in PBS containing 4% paraformaldehyde (2 h; 4°C), decalcified overnight in 0.5 M EDTA (4°C) and cryoprotected in PBS containing 30% sucrose (4°C). The dehydrated VNO was embedded in Tissue Freezing Medium and sectioned at 20 µm on a Leica CM1950 cryostat (Leica Biosystems, Nussloch, Germany).

### Vibratome sections

Acute vomeronasal tissue sections were prepared as previously described (Hagendorf et al., 2009; Spehr et al., 2009). Briefly, mice were sacrificed by brief exposure to CO_2_ followed by decapitation using sharp surgical scissors. The lower jaw and the soft palate were removed allowing access to the vomeronasal capsule. After removal of the cartilage, the dissected VNO was embedded in 4% low-gelling temperature agarose and coronal slices (150–200 µm) were cut in ice-cold oxygenated extracellular solution (**S**_2_) using a Leica VT1000S vibratome (speed: 3.5 a.u. = 0.15 mm/s; frequency: 7.5 a.u. = 75 Hz; amplitude: 0.6 mm; Leica Biosystems). Sections were transferred to a submerged, oxygenated (**S**_2_) and chilled storage chamber until use.

### Immunohistochemistry

Blocking was performed for 1 h in PBS containing 2% goat serum, 1% gelatine and 0.2% Triton X-100 (blocking solution). Sections were then incubated overnight at 4°C with primary antibody sera (1:500 rabbit anti-V2R2; 1:200 rabbit anti-FPR-rs3) in blocking solution, washed in PBS containing 0.05% Triton-X 100 (3 × 10 min, 1 × 30 min), and incubated for 1 h with Alexa^®^ Fluor secondary antibodies (1:500). Excess antibodies were removed by washing in PBS containing 0.05% Triton-X 100 (3 × 10 min, 1 × 30 min). To control for nonspecific staining, experiments in which the primary antibodies were omitted were performed in parallel with each procedure.

### Electrophysiology

Vomeronasal organ slices were transferred to a recording chamber (Luigs & Neumann, Ratingen, Germany) on an upright fixed-stage scanning confocal microscope (TCS SP5 DM6000CFS, Leica Microsystems) equipped with a 20x/1.0 NA water immersion objective (HCX APO L, Leica Microsystems) as well as a cooled a CCD-camera (DFC360FX, Leica Microsystems). Slices were continuously superfused with oxygenated **S**_2_ (~3 ml/min; gravity flow; RT). Patch pipettes (4–7 MΩ) were pulled from borosilicate glass capillaries (1.50 mm OD/0.86 mm ID; Science Products) on a PC-10 micropipette puller (Narishige Instruments, Tokyo, Japan), fire-polished (MF-830 Microforge; Narishige Instruments) and filled with pipette solution (**S**_8_ or **S**_9_) depending on experimental design. An agar bridge (150 mM KCl) connected reference electrode and bath solution. An EPC-10 amplifier controlled by Patchmaster 2.67 software (HEKA Elektronik, Lambrecht/Pfalz, Germany) was used for data acquisition. We monitored and compensated pipette and membrane capacitance as well as series resistance. Only neurons exhibiting small and stable access resistances (≤3% of R_input_; change <20%) were used for analysis. Liquid junction potentials were calculated using JPCalcW software (Barry, 1994) and corrected online. If not stated otherwise, signals were low-pass filtered (analog 3- and 4-pole Bessel filters (–3 dB); adjusted to ^1^/_3_–^1^/_5_ of the sampling rate (5–10 kHz, depending on protocol)). Between recordings, holding potential (V_hold_) was −60 mV. All electrophysiological data were recorded in whole-cell configuration at room temperature.

### Data analysis

All data were obtained from independent experiments performed on at least 3 days using at least three different animals. Individual numbers of cells/experiments (*n*) are denoted in figure legends. If not stated otherwise, results are presented as means ± SEM. Statistical analyses were performed using paired or unpaired *t*-tests or one-way ANOVA with Tukey’s HSD *post hoc* test. Tests and corresponding *p*-values that report statistical significance are individually specified in figure legends. Drug sensitivity of voltage-gated K^+^ (Kv) currents was examined based on an “additive” drug exposure regime, i.e., TEA (1 mM), 4-AP (10 mM), and TEA (25 mM) were sequentially applied and the inhibitor-sensitive currents were isolated by subsequent “offline” subtraction from each preceding recording.

Electrophysiological data were analyzed offline using PatchMaster 2.67 (HEKA Elektronik), IGOR Pro 6.3 (WaveMetrics, Lake Oswego, OR) and Excel (Microsoft, Seattle, WA) software. Activation curves were fitted by the Hill equation to calculate the membrane potential of half-maximal activation (V_1/2_). Current activation time constants (τ) were calculated by fitting individual traces to monoexponential functions *I*_(t)_ = *I*_1_ [exp (−t/τ)] + *I*_0_.

## Results

### Transgenic expression of Fpr-rs3-i-Venus in a subset of neurons in the mouse VNO

**Figure 1:**
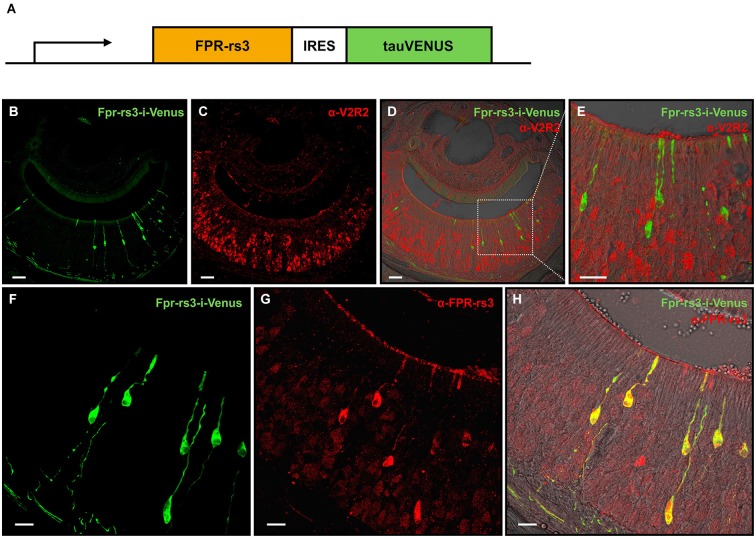
**Generation and characterization of the Fpr-rs3-i-Venus transgenic mouse line**. **(A)** Schematic of the transgene that includes an OR promoter/enhancer, followed by the coding sequence of FPR-rs3 and a polycistron that drives the tau-Venus fluorophore. **(B)** Confocal image of a coronal VNO cryosection showing sparsely distributed fluorescently labeled FPR-rs3^+^ VSNs (green) in the sensory neuroepithelium. **(C)** Confocal image displaying a coronal VNO cryosection immunostained with an antibody against V2R2 (red), a family-C V2R expressed in most basal VSNs. **(D)** Overlay of FPR-rs3 tau-Venus fluorescence and anti-V2R2 staining shows no co-localization of apically located FPR-rs3^+^ and basal V2R2 expressing neurons. **(E)** Higher magnification of the boxed area in **(D)** illustrating the absence of overlapping fluorescence. **(F)** Confocal image of a VNO cryosection showing distinct green fluorescent FPR-rs3^+^ sensory neurons (green). **(G)** Confocal image displaying the same area as in **(F)** stained against the FPR-rs3 protein (red). **(H)** Overlay of tau-Venus fluorescence and antibody staining against the FPR-rs3 protein. Note that all transgene-positive cells also express FPR-rs3 (yellow). Scale bars, 50 µm **(B–D)**, 10 µm **(E)** and 20 µm **(F–H)**.

To analyze the biophysical properties inherent to a prototypical member of the FPR-expressing neurons, we engineered transgenic mice that express Fpr-rs3-i-Venus in a subset of olfactory sensory neurons (OSNs). Using standard transgenic techniques (see section materials and methods), we generated such a mouse strain in which FPR-rs3 is coexpressed with tau-Venus, an enhanced variant of yellow fluorescent protein (Nagai et al., 2002) fused to the microtubule-associated protein tau (Figure 1A). Four founders were obtained. Two of them expressed the transgene in OSNs, and one of them in VSNs. We focused our attention on this latter line, given its exclusive vomeronasal expression pattern. Neither hemi-, nor homozygous Fpr-rs3-i-Venus mice from this line showed any obvious aberrant phenotype.

In coronal VNO tissue slices, a subpopulation of VSNs is fluorescently labeled (81 out of 11,416 neurons (~0.7%); Figures 1B,F) indicating expression of the Fpr-rs3-i-Venus transgene. Fluorescent neurons are morphologically indistinguishable from unlabeled VSNs. Their somata appear to be predominantly located in the apical layer of the neuroepithelium (Figure 1B). Among the five vomeronasal FPRs, FPR-rs3, 4, 6 and 7 are expressed in the more apical G_αi2_-positive layer of the VNO sensory epithelium, whereas FPR-rs1 is located in more basal G_αo_-expressing neurons (Liberles et al., 2009; Rivière et al., 2009). To investigate layer-specific expression of the Fpr-rs3-i-Venus transgene we immunostained coronal VNO cryosections from hemizygous mice with an antibody against V2R2 (α-V2R2; specific for family-C V2Rs that are broadly expressed in the great majority of basal VSNs; Figures 1C–E; Martini et al., 2001; Silvotti et al., 2007). We never observed colabeling of transgene-expressing and V2R2-immunopositive VSNs (*n* = 79) confirming layer-specific expression of the FPR-rs3 transgene in apical VSNs. Immunostaining with an anti-FPR-rs3 antibody (Rivière et al., 2009; Dietschi et al., 2013) revealed 424 out of 53,284 FPR-rs3^+^ VSNs (0.79%), an expression level within the previously reported range between 0.4% and 0.8% (Rivière et al., 2009; Dietschi et al., 2013). Moreover, all transgene-positive cells (*n* = 225) also express the FPR-rs3 protein (Figures 1F–H). Some FPR-rs3-immunopositive neurons (199 out of 424 cells) did not show detectable Venus fluorescence, consistent with the presence of VSNs endogenously expressing FPR-rs3.

### Passive membrane properties of FPR-rs3^+^ VSNs

**Figure 2:**
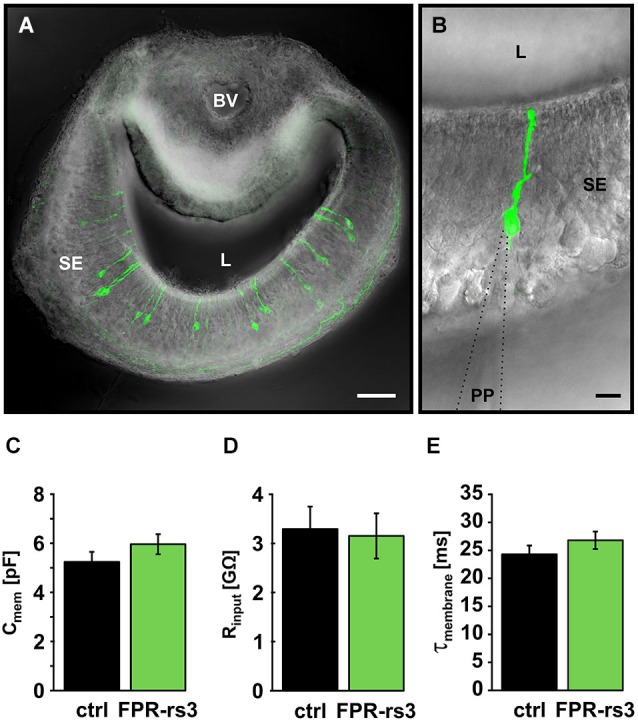
**Passive membrane properties of FPR-rs3^+^ VSNs**. **(A)** Confocal image (maximum projection) of a 150 µm acute coronal VNO tissue slice showing the distribution of fluorescent FPR-rs3 tau-Venus^+^ neurons (green) in the vomeronasal sensory epithelium. Fluorescent axon bundles are visible within the basal lamina. **(B)** FPR-rs3 tau-Venus^+^ neurons exhibit a single apical dendrite ending in a knob-like structure at the luminal border. Whole cell patch-clamp recordings were performed from the VSN soma. **(C)** Membrane capacitance and **(D)** input resistance (R_input_) are similar for both control and FPR-rs3^+^ neurons (*n* = 21).** (E)** Membrane time constant (τ_membrane_) of control neurons compared to FPR-rs3^+^ cells shows no significant difference (*n* = 21). Data are mean ± SEM. Blood vessel (BV), lumen (L), patch pipette (PP), sensory epithelium (SE). Scale bars, 50 µm **(A)**, 10 µm **(B)**.

The passive membrane properties of a neuron determine its basic electrophysiological characteristics and, thus, control its individual stimulus-response function. For FPR-rs expressing vomeronasal neurons, these critical physiological parameters are unknown. Using Fpr-rs3-i-Venus mice, we performed whole-cell patch-clamp recordings from optically identified, fluorescently labeled FPR-rs3-expressing neurons in acute VNO tissue slices (Figures 2A,B). For quantitative comparison, we additionally performed a series of control experiments in randomly chosen VSNs from C57BL/6 wild type mice.

Passive membrane properties (i.e., input resistance (R_input_), membrane capacitance (C_mem_), and membrane time constant (τ_mem_)) were obtained immediately after membrane rupture. Treated, to a first approximation, as a “biological constant” with a value of ~1 µF/cm^2^ (Gentet et al., 2000), C_mem_ was determined using a square pulse (5 mV, 10 ms) routine. Transgene-positive (FPR-rs3^+^) neurons revealed an average C_mem_ value of 5.96 ± 0.49 pF (*n* = 21), similar to data obtained from control VSNs (5.24 ± 0.38 pF; *n* = 21; Figure 2C). We next determined R_input_ at the VSN soma by measuring the steady-state voltage response to a current step of defined amplitude. The average somatic R_input_ of FPR-rs3^+^ neurons was 3.15 ± 0.49 GΩ (*n* = 21; Figure 2D). This large value resembles R_input_ measurements from control VSNs (3.29 ± 0.43 GΩ; *n* = 21), suggesting that FPR-rs3^+^ neurons share the extraordinary sensitivity of V1/2R-expressing VSNs (Liman and Corey, 1996; Shimazaki et al., 2006; Hagendorf et al., 2009). Linear passive voltage responses were also used to estimate τ_mem_ from monoexponential fits to the voltage responses (from onset to steady state). We obtained relatively slow τ_mem_ values of 26.79 ± 2.25 ms (*n* = 21) in FPR-rs3^+^ neurons vs. 24.29 ± 1.57 ms (*n* = 21) in control neurons (Figure 2E).

Together, these results describe different passive membrane parameters of FPR-rs3^+^ neurons. Moreover, these data show that the passive electrical properties of FPR-rs3 expressing VSNs do not significantly differ from control neurons, suggesting (a) that FPR-rs expressing VSNs are not segregated or isolated from the “general” VSN population; and (b) that transgene expression *per se* does not perturb the passive biophysical properties of FPR-rs3^+^ neurons.

### Active membrane properties of FPR-rs3^+^ neurons

**Figure 3:**
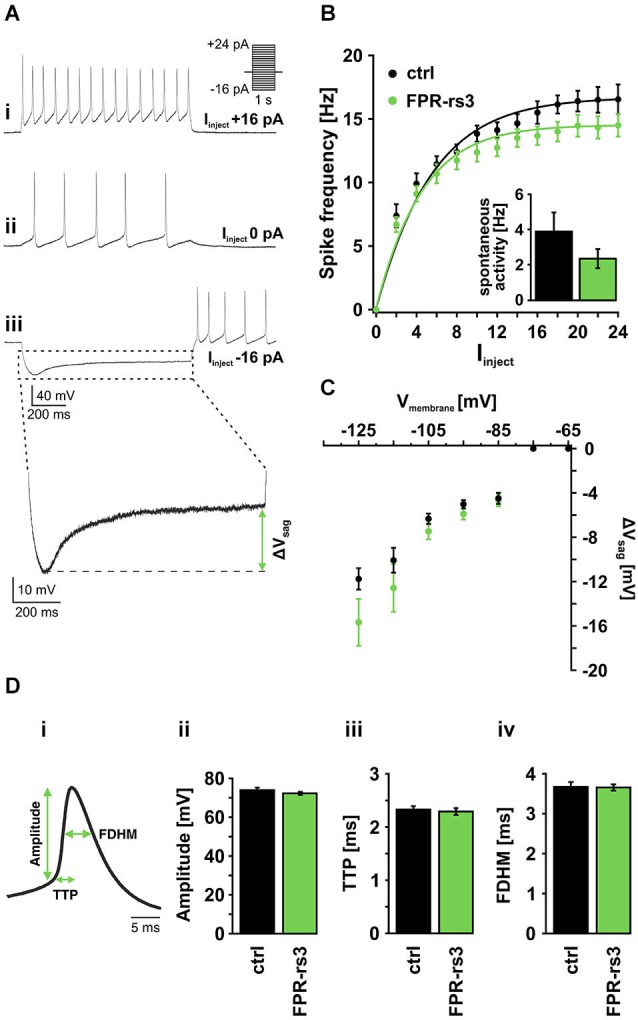
**Active membrane properties of FPR-rs3^+^ VSNs**. **(A)** Representative current clamp traces showing de- / hyperpolarization and trains of (rebound) action potentials generated upon stepwise current injection. Note the spontaneous activity measured at 0 pA current injection **(A_ii_)**. Injection of negative current produces a prominent voltage “sag” most likely mediated by activation of HCN channels **(Aiii)**. **(B)** Firing frequency of control (*n* = 21) and FPR-rs3^+^ (*n* = 19) neurons as a function of the injected current (I_inject_). The gradual increase in firing rate is comparable for control and FPR-rs3^+^ VSNs. Inset: Spontaneous spiking frequency at 0 pA current injection. Note that *f*-I curves have been “background-corrected” using these values. **(C)** Voltage “sag” (ΔV_sag_, *n* = 3–32) as a function of the peak membrane hyperpolarization (10 mV bins). ΔV_sag_ values of control and FPR-rs3^+^ neurons show no statistical difference (*p* > 0.01, two-tailed Student’s *t*-test). **(D)** Average spike waveform illustrating analysis parameters (amplitude, time-to-peak (TTP), full duration at half-maximum (FDHM; **D_i_**). Amplitude analysis of the first action potential for each current injection step shows no difference between control and FPR-rs3^+^ cells **(D_**ii**_)**. TTP analysis reveals values in the same range for both cell populations **(D_iii_**). Spike width (FDHM) is not significantly different between control and FPR-rs3^+^ VSNs **(D_iv_**). Data are mean ± SEM.

Next, we examined the active membrane properties of FPR-rs3^+^ neurons. A hallmark of VSNs is that depolarizing current injection of only a few picoamperes triggers repetitive action potential discharge (Liman and Corey, 1996; Shimazaki et al., 2006). This also holds true for FPR-rs3^+^ neurons (Figure 3A). Current-clamp recordings from fluorescently labeled VSNs show repetitive spiking in response to depolarizing current steps of 2–24 pA. Spontaneous activity (measured at 0 pA current injection) was 2.37 ± 0.54 Hz (*n* = 19) for FPRrs3^+^ neurons and 3.9 ± 1.08 Hz (*n* = 21) for control cells (Figure 3B, inset). By plotting mean instantaneous spike frequencies as a function of stationary current input (*f*-*I* curve; Figure 3B), response saturation at amplitudes >20 pA becomes apparent (maximum frequency *f*_max_ = 14.5 ± 0.88 Hz (*n* = 19; FPR-rs3^+^ neurons) or 16.54 ± 1.17 Hz (*n* = 21; control VSNs)). Injection of negative current into FPR-rs3^+^ neurons revealed a hyperpolarization-activated rebound depolarization (“sag”; Figure 3A_iii_), indicative of I_h_ currents and, thus, HCN channel expression (Robinson and Siegelbaum, 2003; Dibattista et al., 2008). Plotting the sag potential amplitude (ΔV_sag_; Figure 3A_iii_) as a function of peak hyperpolarization reveals the threshold (< −75 mV) and voltage dependence of the sag (*n* = 5–23; Figure 3C), likely corresponding to an increase in HCN channel activation at more negative membrane potentials. A similar voltage dependence was observed for control cells (*n* = 5–32). In both FPR-rs3^+^ and control VSNs, we frequently observed rebound spikes upon repolarization (Figure 3A_iii_).

Next, we examined action potential discharge of FPR-rs3^+^ neurons. Figure 3D_i_ depicts an averaged spike waveform and shows schematically how different spike parameters were analyzed: spike amplitude was measured as the threshold-to-peak distance, spike duration was calculated as the full duration at half-maximum (FDHM), spike generating kinetics was measured as the time-to-peak (TTP). All analyses were based on the first spike of a given train of action potentials (see Figure 3A_i_). Our results reveal an average amplitude of 72.24 ± 0.97 mV (*n* = 134) for FPR-rs3^+^ neurons and 73.92 ± 0.87 mV (*n* = 172) for control neurons (Figure 3D_i_). Average TTP values were 2.29 ± 0.06 ms (FPR-rs3^+^ cells) and of 2.33 ± 0.09 ms (control neurons), while FDHM was 3.65 ± 0.08 ms (FPR-rs3^+^ neurons) and 3.67 ± 0.12 ms (control VSNs), respectively.

These data show that FPR-rs3 expressing VSNs exhibit rather slow action potentials and, albeit an extraordinary sensitivity, show a relatively narrow spike frequency coding range. Together, these active membrane properties are shared by both FPR-rs3 expressing and control neurons.

### Voltage-gated Na^+^ currents of FPR-rs3^+^ neurons

**Figure 4:**
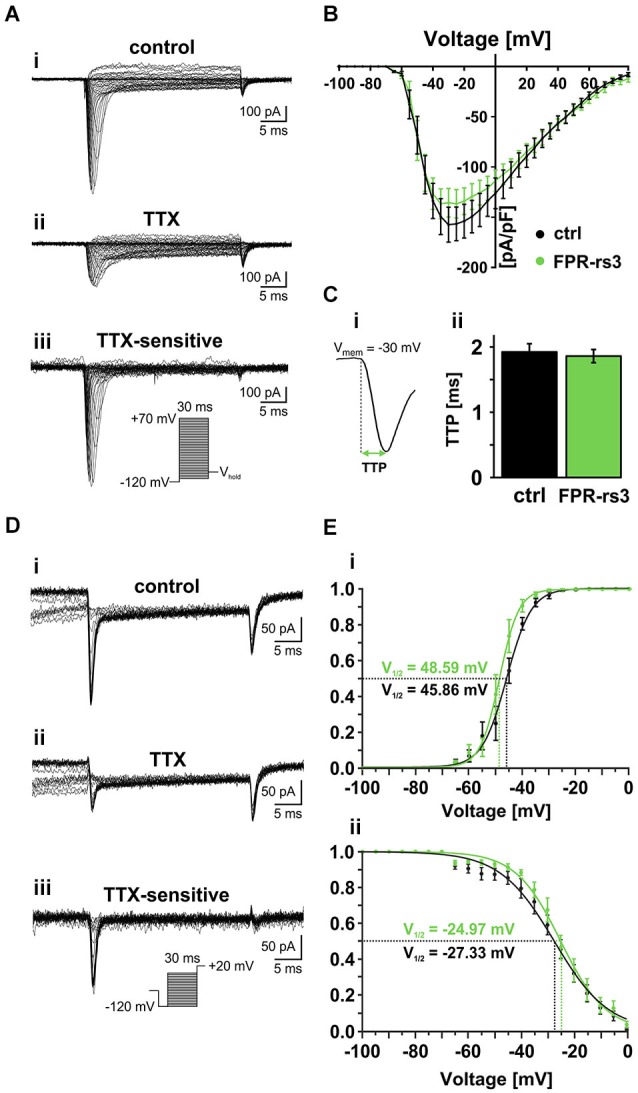
**Voltage-gated Na^+^ currents**. **(A)** Representative traces from whole-cell patch-clamp recordings of a TTX-sensitive fast activating Na^+^ current in FPR-rs3^+^ VSNs. **(A_i_)** Voltage step recording under control conditions (extracellular solution **S_1_**; intracellular solution **S_9_**) reveals a voltage-dependent fast and transient inward current. **(A_ii_)** TTX treatment (1 μM) strongly diminishes the current. Digitally subtracted trace (control—TTX **(A_iii_)**) reveals the TTX-sensitive voltage-gated Na^+^ current. **(B)** Current-voltage relationships of TTX-sensitive Na^+^ currents isolated from control and FPR-rs3^+^ neurons (control, *n* = 20; FPR-rs3^+^, *n* = 10; *p* > 0.01, two-tailed Student’s *t*-test). **(C)** Example of a voltage-clamp recording showing the fast activating transient inward current used for upstroke kinetics analysis **(C_i_)**. TTP of the fast activating Na^+^ current upon depolarization to −30 mV (control, *n* = 20; FPR-rs3^+^, *n* = 10; **C_ii_)**. **(D)** Representative traces showing Na^+^ channel steady-state inactivation under control conditions **(D_i_)**, in presence of TTX **(D_ii_)**, and after digital subtraction (control—TTX **(D_iii_)**). Prepulse steps from −120 mV to 0 mV were applied to analyze inactivation (**D_iii_**, inset). **(E)** Normalized activation **(E_i_)** and steady-state inactivation **(E_ii_)** curves (peak current vs. pulse / prepulse voltage). Data points were fitted using a sigmoidal Boltzmann-type equation. Membrane voltage inducing half-maximal activation and inactivation (V_½_) as indicated. Data are mean ± SEM.

In excitable cells, voltage-gated Na^+^ (Na_V_) channels are primarily responsible for action potential initiation and impulse propagation. Upon membrane depolarization, Na_V_ channels mediate the rapid Na^+^ influx that underlies the upstroke of the action potential. However, the electrophysiological properties of the nine homologous members of the Na_V_ channel family (Na_V_1.1 to Na_V_1.9) are not identical and even small differences in Na_V_ channel expression can have profound effects on electrical excitability (Hille, 2001). Therefore, we next focused on macroscopic voltage-activated Na^+^ currents (I_Nav_) in FPR-rs3^+^ neurons. Stepwise depolarizations from −120 mV to +70 mV (30 ms duration; 5 mV increment) in absence and presence of tetrodotoxin (TTX; Figure 4A_i–ii_; Narahashi et al., 1966; Wu and Narahashi, 1988) allowed pharmacological isolation of the TTX-sensitive I_Nav_ (Figure 4A_iii_). Plotting peak I_Nav_ density as a function of membrane depolarization, the current-voltage relationship (Figure 4B) reveals an activation threshold at approximately −65 mV and a maximum current density of −136.7 ± 14.1 pA/pF (*n* = 10). Similar values were recorded from control VSNs (maximum I_Nav_ = −157.5 ± 17.4 pA/pF; *n* = 20). Figure 4C_i_ illustrates the kinetics of channel gating during a single depolarizing step in membrane potential (−30 mV). As expected from relatively slow action potential firing in FPR-rs3^+^ neurons (Figure 3D), TTP analysis of I_Nav_ reveals relatively slow activation kinetics (1.86 ± 0.10 ms; *n* = 10; Figure 4C_ii_).

Next, we examined the voltage-dependence of TTX-sensitive I_Nav_ activation and inactivation in FPR-rs3^+^ neurons (Figures 4D,E). Fitting normalized peak I_Nav_ amplitudes vs. voltage to a sigmoidal (Boltzmann) function demonstrates half-maximal current activation upon depolarization to approximately −50 mV (V_1/2_ = 48.6 mV; *n* = 9; Figure 4E_i_). Steady-state I_Nav_ inactivation was analyzed upon depolarization to +20 mV, preceded by prepulse steps to different potentials ranging from −120 mV to 0 mV (30 ms duration; 5 mV increment; Figure 4D). Again, offline subtraction of TTX-insensitive currents (Figure 4D_ii_) from control recordings (Figure 4D_i_) allowed pharmacological isolation of TTX-sensitive I_Nav_ (Figure 4D_iii_). Steady-state inactivation curves are derived from inverse sigmoidal fits to normalized peak I_Nav_ amplitudes vs. prepulse voltage (Figure 4E_ii_) and reveal half-maximal inactivation upon depolarization to V_1/2_ = −25 mV (*n* = 10). Interestingly, at voltages ranging from approximately −60 mV to −5 mV, activation and inactivation curves overlap, suggesting coexpression of multiple Na_V_ channel isoforms and/or a substantial “window current”.

Together, these results demonstrate that FPR-rs3^+^ VSNs express one or more Na_V_ channel isoform(s) that exhibit relatively slow activation upon membrane depolarization >−65 mV with half-maximal and complete activation at ~−50 mV and −30 mV, respectively. Moreover, the slope of the steady-state inactivation curve is relatively shallow, revealing that full channel inactivation only occurs at positive potentials. Since all measured parameters are similar to data recorded from control VSNs, our data further substantiate the notion that that FPR-rs expressing neurons do not constitute a biophysically segregated “outgroup” of VSNs.

### Voltage-gated K^+^ currents of FPR-rs3^+^ neurons

To a large extent, Kv channels control electrical signaling in excitable cells. Accordingly, the large and extended Kv channel family is functionally diversified by alternative splicing, oligomeric subunit assembly, and subcellular targeting (Jan and Jan, 2012). As Kv channels are involved in regulating a wide range of neuronal functions, such as setting the resting membrane potential, dictating the duration and/or frequency of action potentials, volume regulation, etc., we next characterized Kv channel-mediated currents (I_Kv_) in FPR-rs3^+^ neurons.

Activated by depolarization, outward flux of K^+^ repolarizes the membrane and, thus, contributes to action potential termination and, in some neurons, afterhyperpolarization. To isolate different classes of I_Kv_ we used a pharmacological toolkit of several well-described Kv channel inhibitors (Alexander et al., 2013). Depending on concentration, tetraethylammonium (TEA) functions as a relatively selective inhibitor of big conductance Ca^2+^-dependent K^+^ (BK) channels at low millimolar concentrations (Yellen, 1984), whereas substantially higher concentrations (25 mM) serve as a nonselective “broadband” Kv channel blocker (Alexander et al., 2013). In addition, 4-aminopyridine (4-AP) specifically blocks A-type K^+^ currents in various neurons (Mei et al., 1995; Amberg et al., 2003).

**Figure 5:**
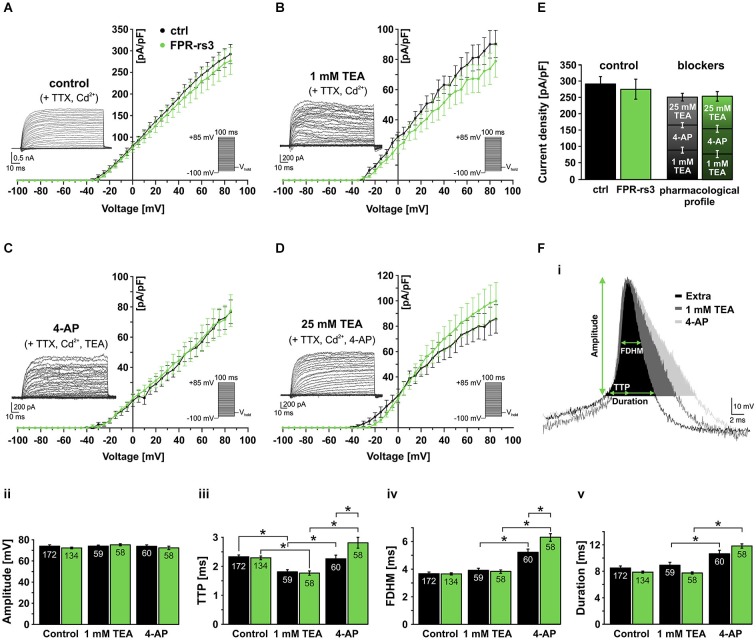
**Voltage-gated K^+^ currents and their role in action potential firing. (A)** Voltage-activated outward K^+^ currents under control conditions (solution **S_1_** including TTX (1 µM) and Cd^2+^ (200 µM) to isolate K^+^ currents). Currents were induced by stepwise depolarization and measured during steady-state. Current densities were calculated and plotted against voltage (control, *n* = 10; FPR-rs3^+^, *n* = 13). **(B)** Outward currents sensitive to 1 mM TEA (control-TEA; digital subtraction; *n* = 10). **(C)** Outward currents sensitive to 10 mM 4-AP (control/TEA-4-AP; digital subtraction; *n* = 10). **(D)** Outward currents sensitive to 25 mM TEA (control/TEA/4-AP-TEA; digital subtraction; *n* = 10). **(E)** Quantification of outward currents. Maximum current densities under control conditions (left bars; 292.5 ± 22.4 pA/pF at +85 mV, *n* = 10 (ctrl); 276.5 ± 31.1 pA/pF, *n* = 13 (FPR-rs3^+^)) and added drug sensitive current densities (ctrl: 90.3 ± 9.1 pA/pF (1 mM TEA), 76.8 ± 7.9 pA/pF (10 mM 4-AP), 85.7 ± 11.2 pA/pF (25 mM TEA); FPR-rs3^+^: 78.9 ± 10.6 pA/pF (1 mM TEA), 77.5 ± 10.5 pA/pF (10 mM 4-AP), 99.8 ± 14.6 pA/pF (25 mM TEA)). Data are mean ± SEM. **(F)** Representative spike waveform under control conditions (solution **S_1_** (Extra)) and in presence of TEA (1 mM) and 4-AP (10 mM; **F_i_**). Analysis parameters (amplitude, TTP, FDHM and spike duration) are depicted schematically. Bar graphs illustrate the quantification of discharge characteristics **(F_ii–v_)**. *p < 0.01; two-way ANOVA with Tukey’s multiple comparisons test. Data are mean ± SEM, number of cells as depicted inside the bars.

Under control conditions, stepwise depolarization from −100 to +85 mV (100 ms duration; 5 mV increment) triggered large outward currents that essentially showed no sign of inactivation (Figure 5A; inset). When steady-state currents were plotted as a function of depolarization, the resulting current-voltage relationship reveals I_Kv_ activation at approximately −30 mV (Figure 5A). Linear regression from data points corresponding to full activation (+60 mV – +85 mV) indicates I_Kv_ reversal at ~−65 mV. When drug-sensitive currents were isolated by digital subtraction of blocker-insensitive from respective “control” recordings (Figures 5B–D, insets; see section materials and methods), the resulting current-voltage plots revealed no statistical differences between FPR-rs3^+^ neurons and control VSNs (Figures 5B–D). Somewhat surprisingly, currents isolated by 4-AP treatment did not show a pronounced transient component typical for A-type K^+^ currents. Interestingly, summation of the individual drug-sensitive I_Kv_ components added up to almost 100% of control currents (276.5 ± 31.1 pA/pF at +85 mV; *n* = 13; Figure 5E) showing that a “cocktail” of 4-AP (10 mM) and TEA (25 mM) is sufficient to block essentially all Kv channels in FPR-rs3^+^ neurons. This pharmacological profile was statistically indistinguishable from control VSNs.

Next, we investigated how the pharmacologically different K_v_ channel populations shape action potential discharge in FPR-rs3^+^ cells. Spikes were elicited and discharge parameters were analyzed as described (Figures 3D, 5F_i_). VSNs were challenged with either TEA (1 mM) or 4-AP (10 mM). Spike amplitude (Figure 5F_ii_) was not altered by either drug. Both Kv channel inhibitors affected the upstroke dynamics (Figure 5F_iii_). However, while block of putative BK channels by TEA (1 mM) accelerated the upstroke, inhibition of A-type currents prolonged the average TTP. 4-AP treatment also prolonged the spike width (FDHM) and, consequently, spike duration (Figure 5F_iv–v_) whereas TEA did not elicit such effects. The effects of 4-AP are significantly more pronounced in FPR-rs3 expressing VSNs than in control neurons (Figure 5F_iii–v_).

In summary, these data demonstrate that multiple Kv channel subunits are expressed in FPR-rs3^+^ neurons. These different channel populations synergistically shape the firing properties of FPR-rs3 expressing VSNs. Moreover, with the notable exception of 4-AP-sensitive channel function during discharge, the Kv channel expression profile of FPR-rs3^+^ neurons is largely comparable to control VSNs.

### Voltage-gated Ca^**2+**^ currents of FPR-rs3^**+**^ neurons

Voltage-gated Ca^2+^ (Ca_V_) channels are integral constituents of a neuron’s Ca^2+^ signaling toolkit (Berridge et al., 2003). As such, they are key signal transducers that transform electrical impulses (depolarization) into a biochemically relevant signal (Ca^2+^ influx) that regulates a wide variety of cellular events (Catterall, 2000b; Clapham, 2007). We therefore investigated Ca_V_ currents (I_Cav_) in FPR-rs3^+^ neurons.

**Figure 6:**
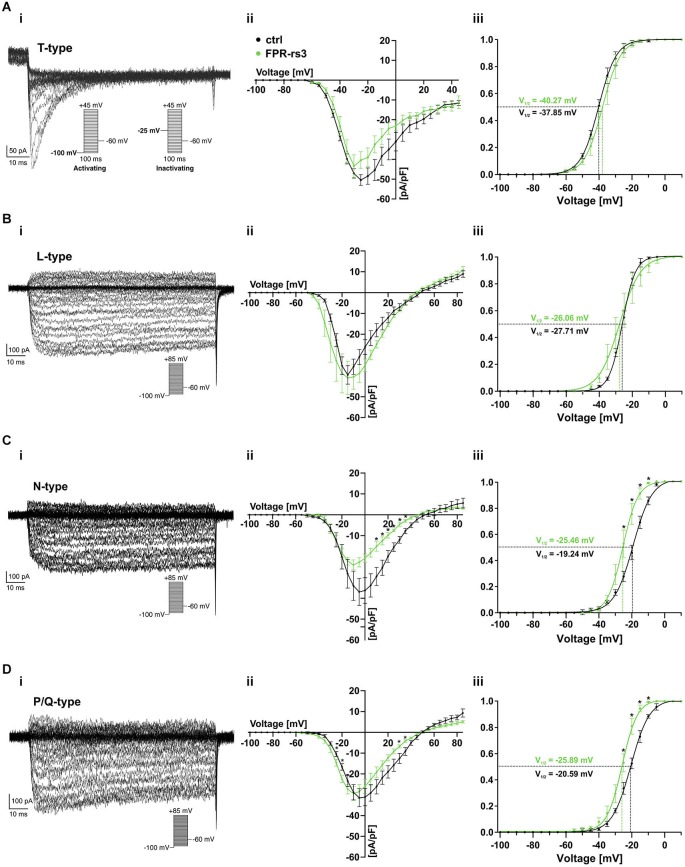
**Voltage-gated Ca^2+^ currents**. **(A–D)** Representative Ca^2+^ current traces isolated either biophysically (prepulse inactivation protocol; **(A_i_)**) or pharmacologically (nifedipine (10 μM; **B_i_)**; ω-conotoxin-GVIA (2 μM; **C_i_)**; ω-agatoxin IVA (200 nM; **D_i_)**). Step protocols as indicated. Absolute **(A_ii_–D_ii_)** and normalized **(A_iii_–D_iii_)** peak current densities are plotted as a function of membrane depolarization. Activation curves **(A_iii_–D_iii_)** are fitted according to a sigmoidal Boltzmann-type equation. Membrane voltage inducing half-maximal activation (V_½_) as indicated. Data are mean ± SEM; *p < 0.01, two-tailed Student’s *t*-test.

The ten functional vertebrate Ca_V_ channel subunits are divided into three subfamilies (Ca_V_1 to Ca_V_3) that differ in function and regulation (Triggle et al., 2006). Both within and between subfamilies, individual Ca_V_ channel isoforms are identified by their distinct biophysical properties and pharmacological profiles (Catterall, 2000b; Alexander et al., 2013). Thus, we isolated transient (T-type) currents mediated by members of the Ca_V_3 subfamily by digital subtraction of I_Cav_ recorded in response to depolarizing voltage steps (−100 mV to +45 mV; 100 ms duration; 5 mV increment) from two different prepulse potentials (−100 mV and −25 mV, respectively; Figure 6A_i_, inset). Based on steady-state inactivation of Ca_V_3 channels at −25 mV (Catterall et al., 2005), the fraction of low voltage activated (LVA) Ca^2+^ channels becomes readily apparent after subtraction (Figure 6A_i_). As expected, these T-type currents rapidly inactivate and the underlying activation and inactivation kinetics become faster with increasing depolarization (Perez-Reyes et al., 1998). The resulting current-voltage relationship (Figure 6A_ii_) and normalized I_Cav_ activation curve (Figure 6A_iii_) demonstrate an activation threshold of −60 mV and half-maximal current activation upon depolarization to −40 mV (V_1/2_ = −40.27 mV; *n* = 9), values typical for T-type currents.

Next, we investigated functional expression of high voltage activated (HVA) Ca_V_ channels in FPR-rs3^+^ neurons. All four members of the Ca_V_1 subfamily are characterized by both long-lasting and large (L-type) Ca^2+^ currents and high sensitivity to dihydropyridines, such as nifedipine (Catterall et al., 2005). Therefore, to examine L-type I_Cav_, we recorded responses to depolarizing voltage steps (−100 mV to +85 mV; 100 ms duration; 5 mV increment) and isolated nifedipine-sensitive currents by digital subtraction (Figure 6B_i_). As expected for L-type currents, isolated I_Cav_ shows relatively slow, though lasting activation upon depolarization ≥ −45 mV (Figure 6B_ii_). Half-maximal activation is observed upon more pronounced depolarization (V_1/2_ = −26.06 mV; *n* = 7; Figure 6B_iii_).

Members of the Ca_V_2 subfamily of HVA Ca^2+^ channels are selectively sensitive to peptide neurotoxins from spider and cone snail venoms (Catterall, 2000a). Using ω-conotoxin-GVIA, we next isolated conotoxin-sensitive N-type I_Cav_ from FPR-rs3^+^ neurons (Figure 6C_i_). N-type currents activate upon depolarizations ≥ −40 mV (Figure 6C_ii_). At approximately −25 mV, N-type I_Cav_ is half-maximally activated (V_1/2_ = −25.46 mV; *n* = 7; Figure 6C_iii_). Surprisingly, recordings from control VSNs reveal substantially larger conotoxin-sensitive currents (Figure 6C_ii–iii_). While T- and L-type I_Cav_ in FPR-rs3^+^ VSNs did not significantly differ from control neurons, maximum N-type current density was −24.05 ± 2.37 pA/pF in fluorescently labeled cells (*n* = 7), but −36.96 ± 6.50 pA/pF in control VSNs (*n* = 8). Moreover, half-maximal activation in controls was shifted to more positive values (V_1/2_ = −19.24 mV; *n* = 8; Figure 6C_iii_).

A slight, though also significant difference between FPR-rs3^+^ and control neurons was observed for P/Q-type Ca^2+^ currents that were pharmacologically isolated using ω-agatoxin IVA (Randall and Tsien, 1995; Catterall, 2011). P/Q-type currents revealed relatively slow activation and slight inactivation. Compared to control recordings, both the current-voltage relationship (Figure 6D_ii_) and the sigmoidal activation curve (Figure 6D_iii_) of P/Q-type I_Cav_ in FPR-rs3^+^ neurons was left-shifted to more negative potentials. Maximum current density, however, did not significantly differ between FPR-rs3^+^ VSNs (−29.50 ± 3.31 pA/pF; *n* = 8) and control neurons (−31.46 ± 4.34 pA/pF; *n* = 5).

In summary, the above data show that FPR-rs3^+^ neurons exhibit a variety of Ca_V_ currents, both LVA and HVA. Since both N- and P/Q-type currents show somewhat different properties in FPR-rs3 expressing VSNs, these two Ca_V_2 channel isoforms might play distinct roles in FPR-rs3^+^ neurophysiology.

## Discussion

For most mammals, the VNO is crucial for intra- and interspecific chemical communication. While the basic biophysical properties of both V1R- and V2R-expressing vomeronasal neurons have been described (Liman and Corey, 1996; Trotier and Døving, 1996; Fieni et al., 2003; Shimazaki et al., 2006; Ukhanov et al., 2007; Hagendorf et al., 2009), VSNs that express members of the recently discovered family of vomeronasal FPR-rs proteins (Liberles et al., 2009; Rivière et al., 2009) remain physiologically unexplored. Here, we describe a transgenic mouse model (Fpr-rs3-i-Venus) in which expression of one member of the FPR-rs family (FPR-rs3) is marked by Venus fluorescence. This mouse strain allows identification and electrophysiological analysis of FPR-rs3-expressing neurons in acute VNO tissue slices. Thus, we provide an in-depth analysis of both passive and active membrane properties, including detailed characterization of several types of voltage-activated conductances and action potential discharge patterns, in fluorescently labeled vs. unmarked vomeronasal neurons. Our results reveal a number of similarities, but also some differences in the basic (electro) physiological architecture of transgene-expressing vs. non-expressing neurons.

Vomeronasal transgene expression in Fpr-rs3-i-Venus mice faithfully recapitulates the punctate apical expression pattern of endogenous FPR-rs3 (Rivière et al., 2009; Dietschi et al., 2013). Furthermore, bicistronic expression of the tau-Venus fusion protein additionally targets the fluorescent marker to axons and axon terminals in the AOB. We therefore propose that Fpr-rs3-i-Venus mice not only provide a useful tool for physiological studies of FPR-rs3^+^ neurons in the VNO (as described here), but also for studies of axon targeting and glomerular innervation in the AOB. While, based on the experimental strategy used here, we cannot exclude that FPR-rs3^+^ VSNs additionally express other vomeronasal receptor genes, this appears unlikely since the negative feedback signal that ensures gene exclusion in apical VSNs is also maintained by exogenous expression of another receptor gene, even an OR (Capello et al., 2009).

The specific biophysical profile of FPR-rs3^+^ VSNs is a critical determinant of their sensory input-output function. Passive membrane properties, such as R_input_, C_mem_ and τ_mem_, are therefore crucial functional descriptors of FPR-rs3^+^ neuron physiology. C_mem_ and dendritic geometry together determine the amplitude of the receptor potential as well as, being inversely proportional, the speed of signal propagation along the dendrite (Gentet et al., 2000). C_mem_ values obtained for FPR-rs3^+^ neurons are broadly consistent with previously reported data (Liman and Corey, 1996; Shimazaki et al., 2006; Ukhanov et al., 2007) and do not differ from values recorded from randomly chosen control VSNs from wild type C57BL/6 mice. The remarkably high input resistance previously reported for VSNs (Liman and Corey, 1996; Fieni et al., 2003; Shimazaki et al., 2006; Dibattista et al., 2008; Sagheddu et al., 2010) is shared by FPR-rs3^+^ neurons. Thus, FPR-rs3-dependent receptor currents of even a few picoamperes will be sufficient to trigger action potential discharge. We therefore propose that the primary signal transduction machinery in FPR-rs3^+^ neurons must be balanced by proper gain/offset control mechanisms to avoid false-positive output. In this context, the rather narrow tuning range of the input-output function of FPR-rs3^+^ neurons (and control VSNs) is noticeable. Frequency coding accommodates spike rates between 0 and ~15 Hz that encode receptor currents ranging to a maximum of ~25 pA (note that the “linear” dynamic range of the *f*-I curve is considerably more narrow). Similar values have previously been reported (Liman and Corey, 1996; Ukhanov et al., 2007). The relatively long τ_mem_ values (~25 ms) we obtained for both FPR-rs3^+^ and control neurons ensure that brief stimulatory events will not generate significant output, in line with the idea that stimulus exchange in the VNO is relatively slow probably allowing prolonged VSN receptor-ligand interaction.

Detailed spike waveform analysis revealed rather slow and broad action potentials in line with previously published results (Shimazaki et al., 2006; Hagendorf et al., 2009). Moreover, hyperpolarizing current injection triggers rebound depolarizations resulting in a pronounced “voltage sag” (Robinson and Siegelbaum, 2003; Dibattista et al., 2008). Mediated by HCN channels, we and others observed increasing “sag” amplitudes with membrane potentials becoming more hyperpolarized (Ukhanov et al., 2007; Dibattista et al., 2008). Thus, active membrane properties of FPR-rs3^+^ neurons do not segregate these neurons from the “general” VSN population.

We used the pufferfish toxin TTX to isolate whole-cell currents mediated by voltage-gated Na_V_ channels. FPR-rs3^+^ VSNs express one or more TTX-sensitive Na_V_ channel isoform(s), i.e., Nav1.1, 1.2, 1.3, 1.4, or 1.7 (Hille, 2001), which exhibit relatively slow activation upon membrane depolarization >−65 mV with half-maximal and complete activation at ~−50 mV and −30 mV, respectively. Notably, the slope of the steady-state inactivation curve is relatively shallow, revealing that full channel inactivation only occurs at positive potentials and, in addition, resulting in a substantial “window current” that ranges from approximately −60 mV to −5 mV.

Similar pharmacological approaches were used to isolate currents mediated by K_V_ and Ca_V_ channels, respectively. At least three different and probably heterogeneous populations of K_V_ channels were identified according to their sensitivity to 4-AP and different TEA concentrations, respectively (Liman and Corey, 1996). Interestingly, while 4-AP-sensitive currents lacked a prominent transient component typical for A-type K^+^ currents (Mei et al., 1995; Amberg et al., 2003), this K_V_ channel population exerted considerable effects on action potential waveform. Moreover, these effects on upstroke kinetics (TTP) and spike width (FDHM/duration) where different between FPR-rs3^+^ neurons and control VSNs. In addition to Na_V_ and K_V_ channels, several types of Ca_V_ channels were identified in FPR-rs3^+^ neurons. T-, L-, N-, and P/Q-type I_CaV_ was isolated, either pharmacologically (L-, N-, P/Q-type) or by prepulse inactivation (T-type). While T- and L-type I_Cav_ in FPR-rs3^+^ VSNs did not significantly differ from control neurons, we find that both N- and P/Q-type currents show somewhat different properties in FPR-rs3 expressing VSNs. We can only speculate about the mechanisms that might link FPR-rs3 expression to altered expression and/or functionality of either N- or P/Q-type Ca_V_ channels. The scope of possible explanations ranges from altered *Cacna1a*/*Cacna1b* transcription by random transgene insertion to direct binding of G_β/γ_ to the α1 subunit of either Ca_V_2 channel (Currie, 2010), complex co-regulation scenarios of, for example, accessory channel subunits (Neely and Hidalgo, 2014), or unknown intrinsic properties of a potential subpopulation of neurons that express FPR-rs3 instead of a “native” receptor. Whatever the mechanistic basis, the interpretation of future experiments will have to take potential physiological differences into account, which could arise from transgenic vs. endogenous expression.

The Fpr-rs3-i-Venus mouse model we introduce and the basic electrophysiological characterization we performed provide a foundation for future functional studies of FPR-rs neurophysiology. In analogy to FPR signaling in the immune system, current concepts of FPR-rs function suggest a role as chemoreceptors for inflammation-associated and pathogen-related compounds (Rivière et al., 2009; Chamero et al., 2011; Bufe et al., 2012). Immune system FPRs are broadly tuned detectors of either host- or pathogen-derived inflammatory signals (Le et al., 2002; Migeotte et al., 2006; He et al., 2014). Somewhat controversial results have been reported on the tuning profile(s) of recombinantly expressed vomeronasal FPR-rs proteins (Rivière et al., 2009; Bufe et al., 2012). Fpr-rs3-i-Venus mice will likely prove useful for studying FPR-rs3-ligand interaction in homologous cells.

## Conflict of interest statement

The authors declare that the research was conducted in the absence of any commercial or financial relationships that could be construed as a potential conflict of interest.

## Footnotes

^1^Available at http://www.stanford.edu/~cpatton/webmaxcS.htm
